# Fistula development after anal abscess drainage—a multicentre retrospective cohort study

**DOI:** 10.1007/s00384-023-04576-6

**Published:** 2023-12-13

**Authors:** Daniel Mark Skovgaards, Helene Perregaard, Christian Bakholdt Dibbern, Andreas Nordholm-Carstensen

**Affiliations:** 1 Digestive Disease Center, Bispebjerg Hospital, University of Copenhagen, Copenhagen, Denmark; 2 Surgical Department, Nordsjællands Hospital, University of Copenhagen, Hillerød, Denmark; 3 The Gastrointestinal Unit, Hvidovre Hospital, University of Copenhagen, Copenhagen, Denmark

**Keywords:** Anal abscesses, Fistula, Crohn’s disease

## Abstract

**Purpose:**

Anal abscesses are common and, despite correct treatment with surgical drainage, carry the risk of developing fistulas. Studies identifying risk factors for the development of anal fistulas are sparse. This study aimed to identify the risk factors for anal fistulas after anal abscess surgery.

**Methods:**

This was a multicentre, retrospective cohort study of patients undergoing acute surgery for anal abscesses in the Capital Region of Denmark between 2018 and 2019. The patients were identified using ICD-10 codes for anal abscesses. Predefined clinicopathological factors and postoperative courses were extracted from patient records.

**Results:**

A total of 475 patients were included. At a median follow-up time of 1108 days (IQR 946–1320 days) following surgery, 164 (33.7%) patients were diagnosed with an anal fistula. Risk factors for developing fistulas were low intersphincteric (OR 2.77, 95CI 1.50–5.06) and ischioanal (OR 2.48, 95CI 1.36–4.47) abscesses, Crohn’s disease (OR 5.96, 95CI 2.33–17.2), a history of recurrent anal abscesses (OR 4.14, 95CI 2.47–7.01) or repeat surgery (OR 5.96, 95CI 2.33–17.2), *E. coli*-positive pus cultures (OR 4.06, 1.56–11.4) or preoperative C-reactive protein (CRP) of more than 100 mg/L (OR 3.21, 95CI 1.57–6.71).

**Conclusion:**

Several significant clinical risk factors were associated with fistula development following anal abscess surgery. These findings are clinically relevant and could influence the selection of patients for specialised follow-up, facilitate expedited diagnosis, and potentially prevent unnecessarily long treatment courses.

## Introduction

Anal abscesses and fistulas represent acute and chronic manifestations of an infected anal gland [1, 2]. These conditions have been recognised and treated since antiquity, and symptoms include daily pain and discharge of blood and/or pus. Studies have shown decreased quality of life in patients with recurring anal abscesses and/or fistulas [3].

The treatment of both acute anal abscess and anal fistulas ranges from straightforward standardised incision and drainage to very complex procedures, depending on their anatomical location. Over the years, a plethora of methods and techniques for managing complex fistulas has been added to the surgical repertoire [4].

The development of fistulas in relation to acute anal abscess is well known, with rates ranging from 26 to 46% [5–8]. However, little is known about the association between fistula development and the initial location of anal abscesses [9]. Endoanal ultrasound (EAUS) has joined MRI of the rectum and anal canal as the gold standard for evaluating anal sphincter pathology and is used for diagnosing and classifying both abscesses and fistulas [10, 11].

Clinical factors that could risk stratify patients with an abscess according to the risk of presenting with an anal fistula at follow-up have been assessed in earlier studies [6, 8, 12, 13]. One-quarter of all patients with Crohn’s disease suffer from Crohn’s-related perianal disease, including anal abscesses and fistulas. This is believed to have a different pathogenesis than cryptoglandular abscess and fistula diseases [9, 14]. Postoperative antibiotic treatment is believed to lower the risk of later fistulising disease, whereas patients with diabetes mellitus or gut-specific bacteria in pus cultures might have a higher risk [9, 15, 16]. On the other hand, neither age, sex or smoking habits have yet been associated with the development of perianal fistulas following an anal abscess [9, 17].

To date, Crohn’s has been the only known clinicopathological factor associated with fistula development following anal abscesses. To improve the treatment of patients with cryptoglandular anal fistulas, such as selection for follow-up, and thereby potentially early diagnosis and intervention, it is essential to identify risk factors for fistula development. Thus, this study aimed to identify the clinical risk factors for developing anal fistulas following surgically treated anal abscess disease.

## Method

This was a multicentre, retrospective cohort study of consecutive adult patients undergoing acute surgery for anal abscesses at two university hospitals in the Capital Region of Denmark in 2018 and 2019. Patients were identified using ICD-10-CM codes for anal abscesses, these being K61.0 to K61.5. Inclusion criteria were all patients above the age of 16 undergoing acute surgery for an anal abscess in the defined period of time. Patients with active fistula with/without seton were excluded. Data on predefined clinicopathological factors were extracted from patient records. These data included surgical notes describing the anatomical location of the abscess, suspicion of fistula, and surgical method, including the use of EAUS perioperatively. Data on specific patient histories were noted, most importantly, a history of anal fistula disease, recurrent abscesses, and inflammatory bowel disease (IBD). Furthermore, data on patient demographics, postoperative course, follow-up, other comorbidities, microbiology including pus cultures, and blood samples were extracted from the patient admission files.

This study was conducted in accordance with the STROBE guidelines for reporting cohort studies [18].

In Denmark, patients suspected for an anal abscess are referred to one of the surgical wards of the country. Clinical examination and, if needed, relevant imaging such as EAUS, CT or MRI are performed, and if anal abscess is suspected, the patient is booked for acute surgery. The abscess is drained according to its anatomical localisation and spread in the perianal spatias according to the skeletal muscle rule [19]. This states that all abscesses that penetrate the external anal sphincter (EAS) or levator ani muscle are to be drained percutaneously. If the abscess is localised medially to the EAS or levator ani, the drainage is performed endoluminally to the anal canal or rectum [20]. In cases of recurrent perianal abscess or fistula suspicion during surgery, the patient is directly referred to specialised follow-up 6 to 8 weeks post surgery. At discharge, patients are informed that non-healing of the wound after 4 weeks warrants further follow-up and, in such cases, they should be referred by their general practitioner. Clinical follow-up is performed by specialised proctologists with training in EAUS. The fistula diagnosis at follow-up is confirmed by EAUS in all cases and supplemental MRI in some cases. In case of fistula diagnosis, the patient is always offered further treatment depending on type and comorbidities. In our experience fistulas diagnosed at follow-up do not spontaneously heal, but need surgical intervention.

### Statistics

Potential differences in demographic and/or clinicopathological variables between patient categories were analysed with the chi-squared test and the Mann–Whitney–Wilcoxon’s tests for categorical and continuous variables, respectively. The same methods were applied to analyse the associations between the variables and the development of anal fistulas at follow-up. Adjustment for potential confounding variables was performed using multivariable logistic regression analysis, and the results are presented as odds ratios (OR) with 95% confidence intervals (95% CI). Only variables likely to be associated with the outcome (*p* ≤ 0.20) in the univariate analyses were included in the multivariable logistic regression analysis. The factors adjusted for, including Crohn’s disease, in the multivariate analysis are listed in Table 2 as variables with *p* ≤ 0.20.

A two-tailed *p* value of 0.05 was used as the level of significance. All statistical analyses were performed using R, version 3.0.1 (Vienna, Austria).

## Results

**Fig. 1:**
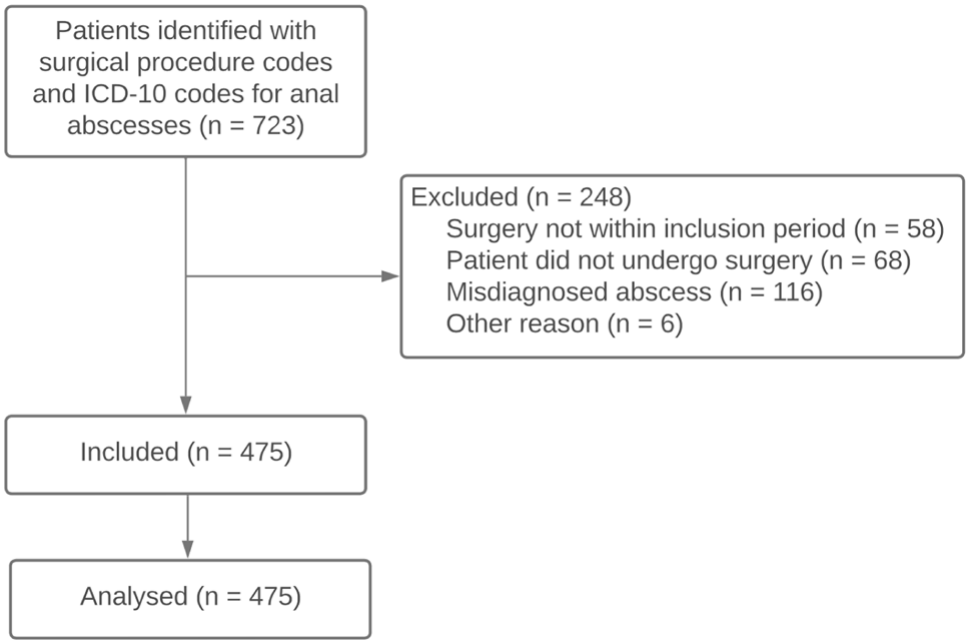
Flowchart showing the patient inclusion flow in the cohort

A total of 723 patients were identified using the ICD-10 codes. Ultimately, 248 patients were excluded because the date of surgery was outside the period of inclusion, they did not undergo surgery, or they had misdiagnosed abscesses (e.g. pilonidal or skin/nates abscesses). Finally, 475 patients were included in the final analysis (Fig. 1). This comprised 475 of 11,312 (4.2%) acute surgeries in the two hospitals during the inclusion period.

In total, 322 of the patients were males (67.8%), median age at surgery was 45 years (IQR 33–58 years) and median BMI was 26.1 kg/m^2^ (IQR 23.0–30.2 kg/m^2^). A history of anal fistula disease was present in 37 (7.8%) patients and 121 (25.5%) had a history of recurrent anal abscess disease. Finally, 25 patients (5.3%) had Crohn’s (Table 1).

**Table 1:** Patient demographics

**Characteristics**	
Patients included, *n*	475
Male sex, *n* (%)	322 (67.8)
Age, years, median (IQR)	45 (33–58)
BMI, median (IQR)	26.1 (23.0–30.2)
Smoking status, *n* (%)	
Never	163 (34.3)
Earlier	83 (17.5)
Active	166 (34.9)
Unknown	63 (13.3)
Alcohol, *n* (%)	
Never	165 (34.7)
Earlier	13 (2.7)
Active	221 (46.5)
Unknown	74 (15.6)
Diabetes, *n* (%)	44 (9.3)
Type 1, *n* (%)	7 (1.5)
Type 2, *n* (%)	37 (7.8)
Inflammatory bowel disease, *n* (%)	29 (6.1)
Crohn’s disease, *n* (%)	25 (5.3)
Ulcerative colitis, *n* (%)	4 (0.8)
Preop. leucocyte count, median, billions/L (IQR)	11.6 (9.1–14.2)
Preop. CRP, median, mg/L (IQR)	30.0 (13.0–64.3)
Known with recurrent abscesses, *n* (%)	121 (25.5)
Known fistula disease, *n* (%)	37 (7.8)

### Anatomical distribution of the abscesses and perioperative EAUS

**Fig. 2:**
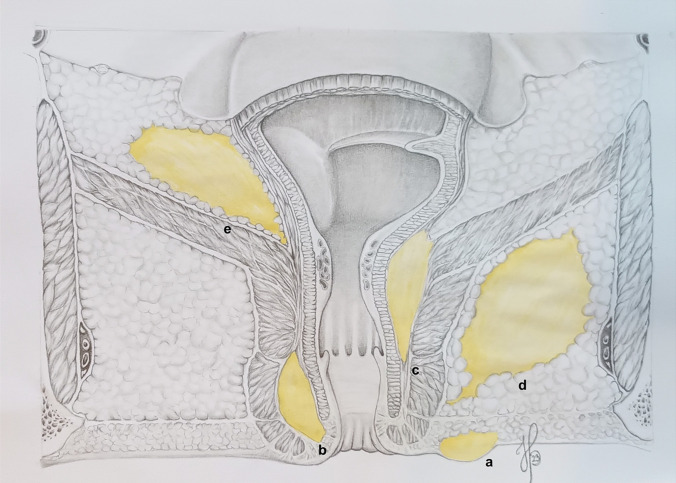
The anatomical definitions of anal abscesses: **a** perianal, **b** low intersphincteric (below the dentate line), **c** high intersphincteric (above the dentate line), **d** ischioanal, **e** supralevator. The illustration is drawn by co-author Helene Perregaard

Figure 2 shows the complete definition of abscess locations (Fig. 2). After locating the abscess according to the anatomy, the precise location was defined by the clock with the patient in lithotomy position. Abscesses were classified as perianal, 59.6% (283 of 475); ischioanal, 14.5% (69 of 475); low intersphincteric, 12.8% (61 of 475); high intersphincteric, 4.0% (19 of 475); and supralevator, 1.0% (5 of 475). A horseshoe abscess was described in 5.0% (25 of 475) of the cases and was located as follows: intersphincteric, 4.0% (19 of 475); ischioanal, 0.8% (4 of 475); and supralevator, 0.2% (1 of 475).

EAUS was performed perioperatively in 137 patients (28.8%). Both centres performed EAUS sporadically when competences were available. The distribution of abscesses in these patients was as follows: perianal, 33.6% (46 of 137); ischioanal, 15.3% (21 of 137); low intersphincteric, 26.2% (36 of 137); high intersphincteric, 9.5% (13 of 137); and supralevator, 1.5% (2 of 137). Horseshoe formation was described in 12.4% (17 of 137), intersphincteric in 10.9% (15 of 137), ischioanal in 0.7% (1 of 137), and one supralevator in 0.7% (1 of 137).

Most abscesses (93.3%, 443 of 475) were surgically drained to the skin. However, 6.5% (31 of 475) were drained endoluminally. Of these, 13 were classified as high intersphincteric, 10 as intersphincteric horseshoe and two supralevator. Only one classified as perianal and two as low intersphincteric were described as drained endoluminally according to electronic patient records. The anatomical localisation of the remaining three abscesses was not stated.

### Perioperative findings and fistula development

A fistula was identified during the primary surgery in 84 of 475 (18.4%) patients. Of these, 47.6% (40 of 84) were treated with a seton and 13.1% (3 of 84) with acute fistulotomy, leaving 41 (48.8%) patients treated solely with abscess drainage. At follow-up, 66 of the 81 (81.5%) fistulas persisted (of these 40 were the ones treated with seton at primary surgery). Thus, 18.5% spontaneously healed.

During surgery, an underlying fistula was suspected but was not identified by the surgeon in 75 of the 475 patients (15.4%). Of these, only 39 (52.0%) had fistulas at follow-up.

Finally, no fistula was suspected in 328 of the 475 patients with acute abscesses. At follow-up of this group, 59 patients (18.0%) had fistulas.

A C-reactive protein (CRP) level > 100 mg/L was found in 64 of the 475 patients. Of these, the majority of the abscesses were classified as high intersphincteric, ischioanal, horseshoe or supralevator (40 of 64, 62.5%). Almost all abscesses were drained to the skin (92.2%) and only six patients were treated with a seton.

### Postoperative data and follow-up

Repeat surgery was performed in 16.9% (82 of 475) of the patients. The reasons for repeat surgery were either planned revision (36.5%, 30 of 82) or insufficient drainage of the abscess at primary surgery (51.2%, 42 of 82). “Planned revision” was ordered by the surgeon after primary surgery typically in cases of large, complex abscesses needing revision in general anaesthesia. Repeat surgery in case of insufficient drainage was planned acutely when the patient did not improve clinically.

In 12.2% (10 of 82) of cases, the reason for reoperation was not specified.

Of the patients who underwent repeat surgery, 31 of 82 (37.8%) had a CRP level > 100 mg/L. In total, five of the 475 (1.0%) patients required a defunctioning stoma. Recurrent abscesses developed in 92 of the 475 patients (18.9%) in the period after surgical treatment. The median number of recurrent abscesses was two (IQR 2–3).

**Fig. 3:**
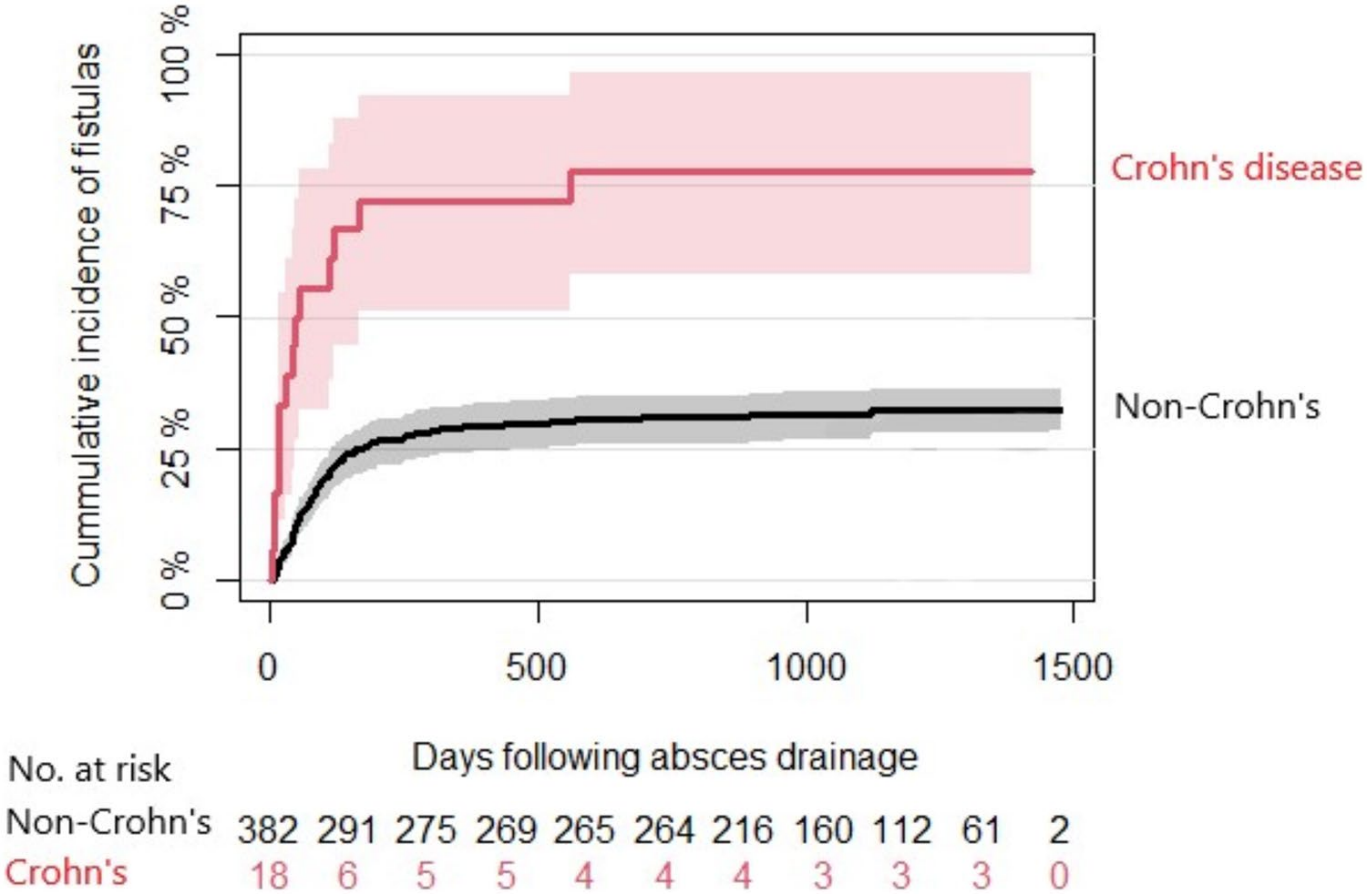
Time to anal fistula diagnosis following anal abscess surgery. The illustration was made in R

The median follow-up period was 1108 days (IQR 946–1320) after surgery. In total, 44.4% (216 of 475) of the patients had clinical specialised follow-up by a proctologist, and 90.3% (195 of 216) underwent EAUS during that consultation. The remaining 259 patients had follow-up by looking in the patient charts. No patients referred to clinical follow-up had loss to follow-up. Unfortunately, data from the MRI scans during the follow-up period were not available. A fistula was found in 34.5% (164 of 475) of all patients undergoing surgery for an anal abscess and in 75.9% (164 of 216) of those who were referred to specialised follow-up by a proctologist. The median time from acute abscess incision to fistula development was 79 days (IQR 41–140 days) for non-IBD patients and 35 days (IQR 14–97 days) for IBD patients (Fig. 3).

Fistulas diagnosed at follow-up were classified as follows: low intersphincteric, 12.2% (20 of 164); high intersphincteric, 5.5% (9 of 164); intersphincteric, not specified, 1.2% (2 of 164); low transsphincteric, 45.2% (74 of 164); high transsphincteric, 22.6% (37 of 164); transsphincteric, not specified, 1.8% (3 of 164); and supralevator, 3.7% (6 of 164). Horseshoe fistula formation was described in nine patients, intersphincteric in 3.1% (5 of 164) and ischioanal in 2.5% (4 of 164). Three fistulas (1.8%) were not classified at follow-up.

### Microbiology

Pus samples were collected from 224 of 475 (47.2%) patients for microbial culture analysis. Of these, 83.5% (187 of 224) were positive for bacteria, with the distribution of different organisms as follows: mixed intestinal flora, 26.8% (60 of 224); *Escherichia coli*, 21.4% (48 of 224); *Bacteroides fragilis*, 7.6% (17 of 224); haemolytic *Streptococcus* group B, 4.0% (9 of 224); *Staphylococcus aureus*, 7.1% (16 of 224); and “other”, 16.1% (36 of 224).

### Univariate and multivariate analyses

In total, 164 of the 475 (34.5%) patients were diagnosed with fistula within the follow-up period. Of these, 29 patients had a history of anal fistula, leaving 135 patients with de novo fistulas. The risk factors for developing fistulas were low intersphincteric (OR 2.77, 95CI 1.50–5.06) and ischioanal (OR 2.48, 95CI 1.36–4.47) abscesses, Crohn’s disease (OR 5.96, 95CI 2.33–17.2), history of recurrent anal abscesses (OR 4.14, 95CI 2.47–7.01) or repeat surgery (OR 5.96, 95CI 2.33–17.2), *E. coli*-positive pus cultures (OR 4.06, 1.56–11.4), or preoperative C-reactive protein (CRP) levels of more than 100 mg/L (OR 3.21, 95CI 1.57–6.71) (Table 2). No differences were found between univariate and multivariate analyses.

**Table 2:** Associations between clinicopathological factors and diagnosis of anal fistula at follow-up, univariate analysis

**Characteristics**	***n/N***	***%***	**OR**^**a**^	**95%** **CI**^**b**^	***p***
Gender					
Male	104/322	32.3	1.00		
Female	60/153	39.2	1.31	0.86–2.02	0.202
Age per 10 years			1.00	0.99–1.02	0.453
Smoking					
Never	68/163	41.7	1.00		
Former	31/83	37.3	1.09	0.62–1.92	0.755
Active	48/166	28.9	0.71	0.43–1.17	0.178
Unknown	17/63	27.0	0.64	0.31–1.23	0.191
Diabetes					
No	155/431	36.0	1.00		
Type I	0/7	0	NA	NA	
Type II	9/36	25.0	0.57	0.22–1.27	0.197
Body mass index					
≤ 20	9/33	27.3	1.00		
> 20–25	50/161	31.1	1.66	0.67–4.73	0.301
> 25–30	62/141	44.0	2.46	0.99–7.03	0.067
> 30	40/114	35.1	1.86	0.73–5.40	0.214
Perioperative EAUS					
No	90/350	25.7	1.00		
Yes	70/137	51.1	2.76	1.78–4.29	< 0.001
Inflammatory bowel disease					
No	141/458	30.8	1.00		
Yes	23/29	79.3	5.96	2.33–17.2	< 0.001
Leucocyte count, 10^9^/L					
> 3–9	38/105	36.2	1.00		
> 9–12	42/139	30.2	0.69	0.39–1.23	0.208
> 12	67/187	35.8	0.93	0.55–1.58	0.778
C-reactive protein, mg/L					
≤ 10	26/81	32.1	1.00		
> 10–50	56/201	27.9	0.99	0.55–1.85	0.981
> 50–100	31/82	37.8	1.50	0.75–3.04	0.255
> 100	33/64	51.6	3.21	1.57–6.71	0.002
Abscess localisation					
Perianal	70/283	24.7	1.00		
Low int	27/61	44.3	3.45	2.01–5.93	< 0.001
High int	7/19	36.8	1.96	0.66–5.30	0.198
Ischioanal	28/69	40.6	2.61	1.46–4.67	0.001
Supralevator	3/5	60.0	5.88	0.95–45.5	0.056
Not specified	6/14	42.9	4.90	1.26–2.04	0.021
History of recurrent abscess					
No	112/362	30.9	1.00		
Yes	52/125	41.6	4.14	2.47–7.01	< 0.001
Reoperation needed					
No	114/409	27.9	1.00		
Yes	50/82	60.1	5.96	2.33–17.2	< 0.001
Microbiology, pus cultures					
No growth	12/37	32.4	1.00		
Mixed intestinal flora	23/60	38.3	1.65	0.64–4.54	0.312
* Escherichia coli*	28/48	58.3	4.06	1.56–11.4	0.005
* Bacteroides fragilis*	6/17	35.3	1.42	0.36–5.31	0.603
Hem. *Streptococcus* gr. B	1/9	11.1	0.39	0.02–2.64	0.408
* Staphylococcus aureus*	3/16	18.8	0.52	0.07–2.49	0.451
Other	11/36	30.6	1.04	0.33–3.27	0.943

^a^*OR* odds ratio

^b^*CI* confidence interval

## Discussion

The present study identified several clinical risk factors that were statistically associated with the diagnosis of anal fistula following anal abscess incision.

At follow-up, 34.5% of the patients undergoing acute surgery for an anal abscess had a fistula. Earlier studies have reported similar rates [5, 6, 17].

Based on the anatomical localisation of anal abscesses, fistula development was significantly associated with low intersphincteric and ischioanal abscesses. Knowing the complexity and nature of high intersphincteric and supralevator abscesses, they are also expected to be associated with complex fistula disease. However, due to their rarity, only a few of these abscesses were included in this study. In a large retrospective cohort comprising 158,713 patients, Sahnan et al. determined the incidence of acute anal abscesses in England [17]. They found that ischioanal and intersphincteric abscesses were associated with fistula formation. However, the intersphincteric abscesses were not differentiated into low/high, and supralevator abscesses were not considered.

As expected, IBD was a significant risk factor for subsequent fistula formation. Almost 80% of the patients with Crohn’s disease or ulcerative colitis (UC) developed anal fistulas. Only four of the 29 patients with IBD had UC, while the remainder had Crohn’s disease. In the study by Sahnan et al., half of the patients with Crohn’s disease developed a fistula after first time admission with an acute anal abscess [17]. This supports the notion that anal abscesses in patients with Crohn’s are likely to originate from or progress into a perianal fistula.

CRP count of 100 mg/L or above on admission to the hospital was significantly associated with a persistent fistula at follow-up. CRP levels are known to increase in response to injury, infection and inflammation [21]. Most of these patients were, not surprisingly, diagnosed with complicated abscesses classified as high intersphincteric, ischioanal, horseshoe and supralevator—but few of them were treated with a seton at primary surgery. Interestingly, an increased white blood cell count was not associated with fistula development. Repeat surgery is fairly frequent when managing anal abscesses, although an association with fistula development has never been detected [22]. In this study, repeat surgery was highly associated with persistent fistulas at the follow-up. This was either a planned revision at the primary surgery or insufficient drainage was detected during the first postoperative days. Of all repeat surgeries, 37.8% had a CRP level > 100 mg/L upon hospital admission. This accounted for half of the patients with a CRP level of > 100 mg/L in the cohort and could support the notion that severe inflammation and reoperation are valid predictors of fistula formation.

Positive pus cultures with *E. coli* growth were also significantly associated with persistent fistulas at follow-up. In a retrospective analysis of patients with acute anal abscesses, Alabbad et al. reviewed the microbiological data of 211 patients with anal abscesses and found *E. coli* to be the most commonly isolated microorganism. In the present study, the most commonly isolated microorganism was also *E. coli* [23]. To our knowledge, no previous study has reported an association with later fistula development [12, 24]. However, the question remains whether it is the bacteria that cause fistulation to persist or whether it is a finding from an established fistula with acute infection.

Unsurprisingly, a history of recurrent anal abscesses was associated with persistent fistula at follow-up. Considering the pathophysiology of the abscess-fistula continuum of acute and chronic inflammation and the evolution of an abscess into a fistula, this makes sense [25]. In this study, one-fifth of the patients with acute abscesses had a fistula diagnosed during primary surgery. Half of these patients were treated only with abscess drainage (no seton or acute lay-open), and at follow-up, approximately one-fifth of them did not have a fistula and were spontaneously healed. When a fistula was only suspected during the primary surgery, only half of the patients were diagnosed with a fistula at follow-up. This could partly be the result of inexperienced surgeons examining the patients and performing the incision, but it also raises the question of how many fistulas heal spontaneously after acute drainage of an anal abscess.

The performance of EAUS during the primary surgery was significantly associated with anal fistula at follow-up. Considering these patients alone, the distribution of the anatomical localisation of the abscesses changed. The proportion of abscesses found in the perianal space was halved, whereas both low and high intersphincteric abscesses more than doubled. This likely represents a selection bias towards the use of EAUS in more complex patients in whom a fistula, complex abscess, or more difficult anatomy is suspected. In everyday clinical practice, this often leads to the procedure being performed by more experienced surgeons with better knowledge of anal anatomy, possibly explaining the change in abscess distribution. Of the patients who underwent EAUS during primary surgery, 44.4% were referred for follow-up and 75.9% of the referred patients had a fistula. Patients suspected of having complex abscess or fistula prior to surgery may have been selected for perioperative EAUS by more experienced surgeons. Thus, occult fistulas are detected at the time of abscess drainage, and many can persist until follow-up. This strengthens the role of ultrasound diagnostics not only in fistula disease in an elective setting but also in patients admitted with acute anal abscesses. In two thorough and recent statements from Italy and Germany, Amato et al. and Ommer et al. reported that 3D-EAUS and MRI, used in combination or alone, were of high and equal quality for diagnosing anal abscesses and fistulas [11, 26].

**Fig. 4:**
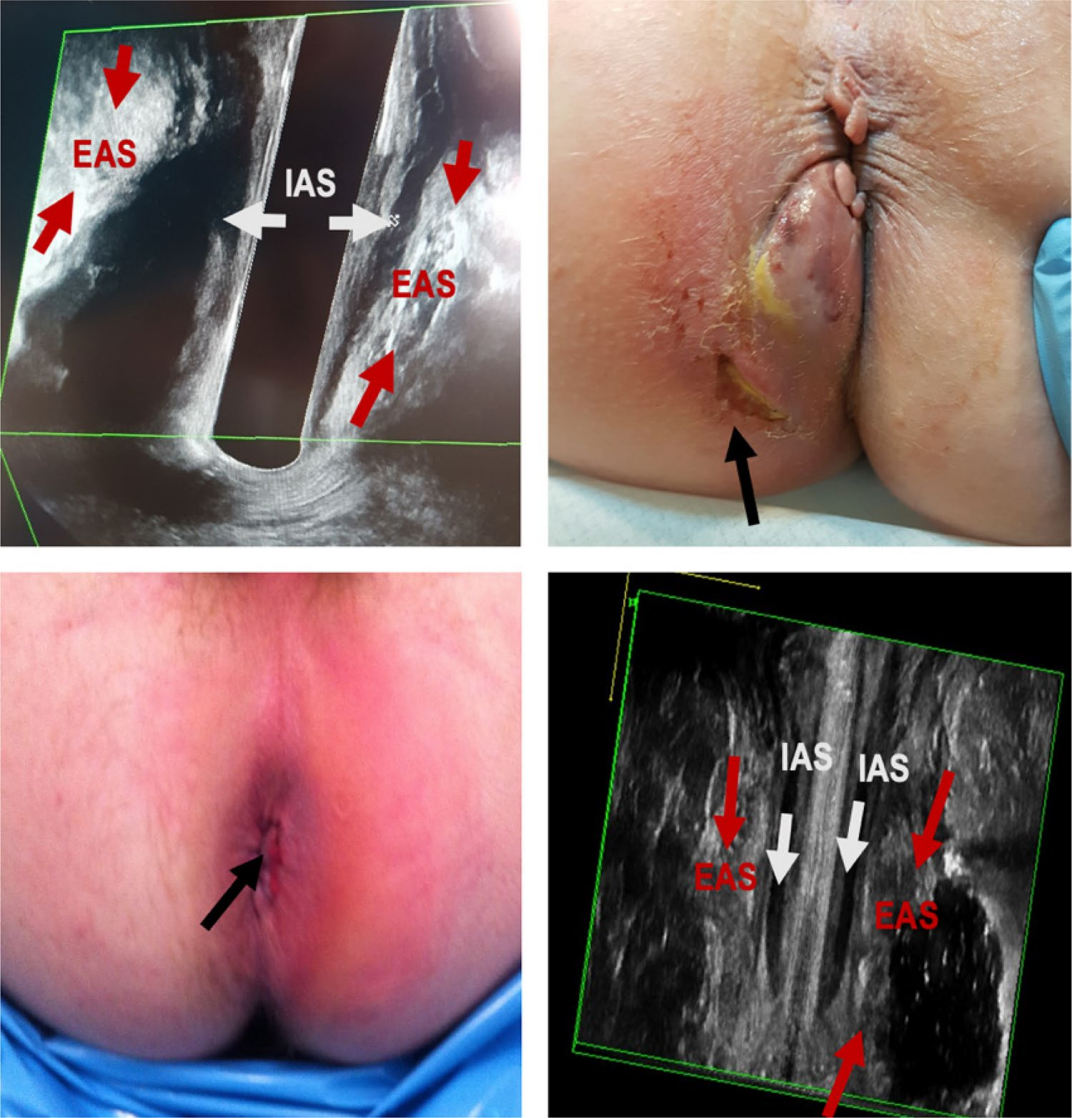
The importance of perioperative EAUS. Top left: EAUS, coronal view of low intersphincteric abscess. Top right: patient with low intersphincteric abscess with attempt of drainage outside of the anal sphincter complex. Bottom left: patient with ischioanal abscess with attempt of drainage via the intersphincteric groove. Bottom right: EAUS, coronal view of ischioanal abscess

In this study, most of the abscesses drained endoluminally and, therefore, often guided by EAUS, were classified as high intersphincteric, supralevator, or with an intersphincteric/supralevator horseshoe formation. These anatomical localisations of abscesses are complex and, if not handled by an experienced surgeon with EAUS, lead to a risk of iatrogenic fistula formation (Fig. 4) [25–28]. Patients not selected for EAUS in the acute setting are often managed by junior staff without sufficient experience in correctly classifying abscesses based solely on physical examinations. This could also affect the type of drainage performed, especially endoanal drainage, which may lead to iatrogenic damage and fistula formation.

The authors acknowledge the limitations of the present study. As with other retrospective studies, this study can only demonstrate associations, not causality. Missing data were inevitable and contributed as an essential limitation of this study. Another important limitation was the quality of the data regarding the anatomical localisation of the abscesses. Patient records were scrutinised for these data, but were often limited by less standardised or poorly specified operation notes. This can lead to incorrectly classified abscesses. As described by Sahnan et al., perianal abscess incisions are often performed by junior doctors, and thus insufficient knowledge of perianal spatias and anatomy is a common problem, and “perianal” is used as an umbrella term in many abscess cases instead of more detailed classifications like “ischioanal, low intersphincteric”. A sampling bias was also considered in this study. Patients were recruited from two hospitals in the same region of one country. This may have affected the generalisability of the results.

In this study, several significant clinical risk factors were associated with the diagnosis of anal fistula at follow-up after surgery for anal abscess. This included specific anatomical locations (low intersphincteric and ischioanal), IBD, a CRP count > 100 mg/L, need for repeat surgery, *E. coli* detected in pus cultures, and patients selected for perioperative EAUS. Despite these limitations, this study of a large cohort contains important and clinically relevant findings that could influence the selection of patients for specialised follow-up with a proctologist. Creating more practical surgical guidelines pertaining to treatment and follow-up after acute anal abscesses could facilitate faster fistula diagnosis and possibly prevent unnecessarily long treatment courses with diminished quality of life.

## Data Availability

On reasonable request.

## Declarations

### Permission to reproduce

On reasonable request.
